# Chronic disease prevalence and care among the elderly in urban and rural Beijing, China - a 10/66 Dementia Research Group cross-sectional survey

**DOI:** 10.1186/1471-2458-9-394

**Published:** 2009-10-21

**Authors:** Zhaorui Liu, Emiliano Albanese, Shuran Li, Yueqin Huang, Cleusa P Ferri, Fang Yan, Renata Sousa, Weimin Dang, Martin Prince

**Affiliations:** 1 Institute of Mental Health, Peking University, Key Laboratory of Mental Health, Ministry of Health (Peking University), No 51 Hua Yuan Bei Road, Haidian District, Beijing 100191, PR China; 2 King's College London, Section of Epidemiology, Health Services & Population Research, De Crespigny Park, London SE5 8AF, UK

## Abstract

**Background:**

Demographic ageing is occurring at an unprecedented rate in China. Chronic diseases and their disabling consequences will become much more common. Public policy has a strong urban bias, and older people living in rural areas may be especially vulnerable due to limited access to good quality healthcare, and low pension coverage. We aim to compare the sociodemographic and health characteristics, health service utilization, needs for care and informal care arrangements of representative samples of older people in two Beijing communities, urban Xicheng and rural Daxing.

**Methods:**

A one-phase cross-sectional survey of all those aged 65 years and over was conducted in urban and rural catchment areas in Beijing, China. Assessments included questionnaires, a clinical interview, physical examination, and an informant interview. Prevalence of chronic diseases, self-reported impairments and risk behaviours was calculated adjusting for household clustering. Poisson working models were used to estimate the independent effect of rural versus urban residence, and to explore the predictors of health services utilization.

**Results:**

We interviewed 1002 participants in rural Daxing, and 1160 in urban Xicheng. Those in Daxing were more likely to be younger, widowed, less educated, not receiving a pension, and reliant on family transfers. Chronic diseases were more common in Xicheng, when based on self-report rather than clinical assessment. Risk exposures were more common in Daxing. Rural older people were much less likely to access health services, controlling for age and health. Community health services were ineffective, particularly in Daxing, where fewer than 3% of those with hypertension were adequately controlled. In Daxing, care was provided by family, who had often given up work to do so. In Xicheng, 45% of those needing care were supported by paid caregivers. Caregiver strain was higher in Xicheng. Dementia was strongly associated with care needs and caregiver strain, but not with medical helpseeking.

**Conclusion:**

Apparent better health in Daxing might be explained by under-diagnosis, under-reporting or selective mortality. Far-reaching structural reforms may be needed to improve access and strengthen rural healthcare. The impact of social and economic change is already apparent in Xicheng, with important implications for future long-term care.

## Background

China, with over 1.3 billion citizens is the world's most populous country [1]. Demographic ageing is occurring at a rate unprecedented for any world region; the proportion of Chinese aged 65 and over will increase from 4% in 2000 to 14% by 2025, amounting to 200 million older people [2]. The prevention and control of chronic diseases is recognised as an urgent priority in China [3,4]. The long term care needs of dependent older people has, comparatively, received much less attention [5-7]. A little over half of China's population lives in rural settings, [8] the proportion among older people is likely to be higher.

The literature on rural/urban differences in health in China suggests three main areas for further study. First, there may be differences in chronic disease prevalence and mortality. Data from the China Health and Nutrition survey showed a 30% increased mortality for those aged 50 and over in rural compared with urban areas, partly mediated through the lack of amenities and lower wages in rural areas [9]. However, in the third Chinese National Health Service Survey (CNHSS), [10] the prevalence of self-reported physician diagnoses of chronic diseases was lower among rural than urban residents. In the 1991 Beijing Longitudinal Study on Aging (BLAS), [11] urban residents were 3.2 times more likely to report chronic disease diagnoses, 4.5 times more likely to report diabetes and 2.5 to three times more likely to report respiratory disease, heart disease and hypertension. On the other hand, levels of disability were similar in the CNHSS, [10] and considerably higher in rural areas in BLAS [11]. Hypertension, when measured directly as opposed to by self-report, is more prevalent in rural areas, [11] and awareness is higher in urban areas [12]. Hence, rural communities seem to be advantaged with respect to some health outcomes, and disadvantaged in others. More research is required to confirm these findings, and to clarify the reasons for the apparent discrepancies.

Second, in China, financing, coverage and access to healthcare depends, largely upon where you live. Only 61% of rural residents, compared with 82% of urban dwellers can access health services within one kilometre of their homes [13]. In urban China, there are two employee-based health insurance schemes, one for government and the other for public and private company employees. There is limited cover for dependents, based on a personal annual subscription. Discounts are available for poor people, those with mental disorders, and retirees. In rural China, the government contributes to a common fund which covers healthcare costs but only proportionate to the amount contributed. In 2003, 79% of rural and 45% of urban residents did not have meaningful health insurance [12]. Almost 50% of health care costs are covered by out-of-pockets payments [14] and more than 35% of urban and 45% of rural households cannot afford any health care [15]. The consequences of these disparities for rural and urban older people, in terms of their ability to access healthcare and to manage their chronic diseases, need to be determined.

Third, social protection (encompassing the range of formal and informal mechanisms to provide safety nets and support to poor and disadvantaged members of society) is under threat for older people in China, as its population ages rapidly. In traditional societies social protection is provided by the family and community. With modernisation, these responsibilities are shared by wider society through intergenerational transfers legislated for and managed by the state [16]. However, in the future there will be fewer children available to provide support and care because of the one child policy [17], and social protection by the state, both in terms of pension coverage and insurance against catastrophic healthcare expenditure, remains very patchy [18]. It is important to understand how these processes may be playing out with respect to older people living in urban and rural communities.

In summary, the urban bias of public policy is particularly marked in China, and older people living in rural areas may be especially vulnerable. The Research Agenda on Ageing Project [19] has advocated more research on this group, including demographic and migration patterns; social transitions; family exchanges; health behaviours and use of and access to healthcare. In this paper we seek to pursue this agenda by

a) comparing the socio-demographic and health characteristics of representative samples of older people in two Beijing communities, urban Xicheng and rural Daxing.

b) describing the patterns of recent health service utilization among rural and urban elderly and estimating the independent effect of health conditions, socio-demographic and socioeconomic factors on access to and use of these services.

c) comparing the levels of disability and needs for care, informal care arrangements and extent of carer strain with respect to dependent older people in urban Xicheng and rural Daxing.

## Methods

### Study Design and catchment areas

This study is part of the 10/66 Dementia Research Group's multi-site international programme on dementia and ageing in low and middle income countries. The full study protocol and procedures are available in an open access online publication [20] From January 2004 to April 2005 we carried out one-phase cross-sectional surveys of all older people (aged 65 years and over) residing in two defined catchment areas: the urban district of Xicheng in the centre of Beijing, close to Tiananmen Square and the 14 villages of Daxing, a rural district 40 kilometres away. In China, rural regions are divided into four classes mainly according to income level, with Class I being the richest and Class IV the poorest [21] - Daxing in common with other rural areas of Beijing is classified under Class I. Participants were identified by means of door-knocking and no exclusion criteria were applied. Sample sizes of 1,000 would allow an estimation of a typical 4.5% dementia prevalence with a standard error of 1.2%. Community doctors administered the interviews, lasting two to three hours, in participants' homes. Informed consent was obtained from informants and participants. The institutional review boards of the Institute of Psychiatry, King's College London and the Institute of Mental Health, Peking University approved the project.

### Measures

The 10/66 protocol comprises questionnaires on participants' sociodemographic characteristics, health status, risk factor exposures and health service use. A physical examination was carried out and an informant interview administered for information on care arrangements, and caregiver practical, psychological and economic strain. All measures and assessments of the 10/66 protocol are described in detail elsewhere, [20] only those relevant for the purposes of this analysis are described here:

1) Sociodemographic characteristics: gender, age, education, marital status, household living circumstances, sources of income (pension and family support), number of household assets

2) Health Status:

a. Chronic disease diagnoses established through structured clinical interviewing and or physical examination. Dementia - all those who on the basis of cognitive testing, clinical and informant interview met either or both of the cross-culturally validated 10/66 dementia [22] or DSM IV dementia criteria [23]. Depression - ICD-10 depressive episodes, ascertained using the Geriatric Mental State structured clinical interview [24]. Self-reported stroke, ischemic heart disease and diabetes ("have you ever been told by a doctor that you had a stroke/heart attack/angina/diabetes?"). Chronic obstructive pulmonary disease, defined as reporting a chronic cough with production of sputum for three or more months. Hypertension - all those with self-reported hypertension ("have you ever been told by a doctor that you have high blood pressure?") and/or a blood pressure measurement meeting the European Society of Hypertension criteria [25]. (systolic blood pressure > = 140 mm Hg and/or diastolic blood pressure > = 95 mm Hg) were considered to have hypertension. Those with self-reported hypertension were considered to be detected. Those with self-reported hypertension but not meeting ESH criteria were considered to be controlled. Those with self-reported treatment ("Were you started on treatment?") were considered to be treated.

b. Physical impairments - self-reported: arthritis or rheumatism; eyesight problems; hearing difficulties or deafness; persistent cough; breathlessness, difficulty breathing or asthma; heart trouble or angina; stomach or intestine problems; faints or blackouts; paralysis, weakness or loss of one leg or arm; skin disorders [26]. Each impairment was rated if it interfered with activities 'a little' or 'a lot'.

c. Disability - measured using the 12 item World Health Organization Disability Assessment Schedule (WHODAS II., developed by WHO for cross-cultural research [27])

d. Risk Factors: Smoking ("ever smoked", "current smoker", "life-time smoking"), hazardous alcohol use ("ever been a heavy drinker" currently and before the age of 60), physical activity ("have you walked at least 0.5 Km in the last month?"), obesity (direct waist circumference in cm and meets waist circumference criteria for metabolic syndrome)

3) Health Service Utilization: Any use of community services (primary care, hospital outpatient, private doctor, dentistry) and hospital admission during the three months preceding the interview.

4) Dependency (needs for care): we used a series of open-ended questions addressed to a key informant, to define the family network, to establish if the older person needed and received care from family members or others and to identify who was responsible for organising and providing 'hands on' care. On the basis of these questions, the interviewer coded whether the older person required no care, care for some of the time or care for much of the time.

5) Care arrangements and impact of providing care

a. Informal care arrangements (time spent with the participant, and time spent assisting to communicate, to use transport, to dress, to eat, and for personal hygiene), paid day and night care

b. The gender of the main carer and their relationship to the cared for older person

c. Carer strain. Carer perceived strain was assessed using the Zarit Burden Interview (ZBI) [28] with 22 items that assess the carer's appraisal of the impact their involvement has had on their lives. The ZBI has been widely used in the USA and Europe, but also in Nigeria and Taiwan [29,30] and in Japan, where it was formally validated [31]. When used in the 10/66 Dementia Research Group pilot studies in 24 centres in Latin America, India, China and Africa the ZBI was found to be practical, culturally relevant, and to have robust psychometric properties [32]. Carer psychological strain was assessed using the 20 item Self-Reporting Questionnaire [33]. Economic strain was assessed according to whether the carer had had to cut back on work to care.

### Analysis

We describe and compare the characteristics of the urban and rural samples according to sociodemographic circumstances (χ^2 ^and t-test, as appropriate), health status (Poisson regression with robust prevalence ratios controlling for age and gender, and for WHODAS II scores negative binomial regression) and health service utilisation (Poisson regression controlling for age, gender and physical impairments).

1) We compared (χ^2 ^tests) the proportion of all hypertension cases that are detected, the proportion of detected cases that are a) treated and b) controlled and the proportion of all cases that are controlled.

2) We estimated the independent predictors of health service utilization (accessing any community health service in the last three months) separately for urban and rural samples. Crude and adjusted prevalence ratios (PRs) with 95% confidence intervals were calculated using Poisson regression adjusting for household clustering, because sometimes two participants of a spouse pair were recruited to the survey. These two persons could not be regarded as statistically independent [34]. Variables included in the model were: dementia diagnosis, one or more physical impairments, age, gender, education, marital status, assets, health insurance and pension.

3) Dementia has previously been shown to be an important determinant of the level and type of informal care, and the extent of carer strain [35,36]. Therefore, among those needing care we assessed the effect of dementia diagnosis, and the effect of rural or urban residence (adjusting for age, gender and independently of dementia status) upon: disability (WHO-DAS II) and dependency, time spent assisting with activities of daily living (ADL), carer strain (ZBI scores) and mental health (SRQ-20), and care arrangements (daytime or night time paid carer, cutting back on work to care, additional informal care). To contrast rural and urban samples, we calculated prevalence ratios (PRs) using Poisson regression adjusting for household clustering for dichotomous outcomes, rate ratios using ordinal regression adjusting for household clustering for ordered categorical variables, and mean differences using General Linear Modelling adjusting for household clustering for continuous variables, all with 95% confidence intervals controlling for age, gender and dementia status.

All analyses were carried out on release 1_5 of the 10/66 dataset using STATA 9.2 (*Stata 9.1*; *Stata *Corp., College Station, Texas)

## Results

In all, 2162 (1160 urban and 1002 rural) participants completed the survey, with 95.7% responding in rural Daxing and 74.3% in urban Beijing, where more eligible people refused to participate or could not be contacted after at least four attempts. The urban elderly were better educated and older, and less likely to be widowed than their rural counterparts (Table 1). Living alone was unusual in either setting, but urban residents were more likely to be living with their spouse only, and less likely to be living with children. Pension coverage was much lower in the rural (3.8%) than the urban sample (90.5%). Conversely, family transfers and rental income were much more common in the rural sample. Only nine urban participants and one rural participant reported receiving a disability pension.

**Table 1:** Social-demographic characteristics in Daxin (rural) and Beijing (urban)

	**Urban (n = 1160)**	**Rural (n = 1002)**	***χ*^2 ^/*Z***	***df***	***P***
**Age group **(MV)	0	0			
65-69 years	316(27.2%)	383(38.2%)	40.4	3	< 0.001
70-74 years	362(31.2%)	296(29.5%)			
75-79 years	254(21.9%)	202(20.2%)			
80+ years	228(19.7%)	121(12.1%)			
**Gender **(MV)	0	0			
Female	661(57.0%)	556(55.5%)	0.5	1	0.49
**Education level **(MV)	0	0			
No education	232(20.0%)	579(57.8%)	491.9	3	< 0.001
Primary education only	456(39.3%)	373(37.2%)			
Completed secondary	335(28.9%)	45(4.5%)			
Completed tertiary	137(11.8%)	5(0.5%)			
**Marital status **(MV)	0	0			
Never married	3(0.3%)	22(2.2%)	52.0	3	< 0.001
Married or cohabiting	829(71.5%)	585(58.4%)			
Widowed	326(28.1%)	394(39.3%)			
Divorced or separated	2(0.2%)	1(0.1%)			
**Previous occupation^1 ^**(MV)	1	1			
Professional/Managerial/Clerical	518(45.0%)	37(3.7%)	1888.6	3	< 0.001
Skilled or semi-skilled	421(36.5%)	14(1.4%)			
Unskilled	207(18.0%)	13(1.3%)			
Agricultural worker	7(0.6%)	938(93.6%)			
**Living arrangements **(MV)	0	0			
Alone	54(4.7%)	49(4.9%)	202.0	3	< 0.001
With spouse only	415(35.8%)	194(19.4%)			
With children	446(38.4%)	679(67.8%)			
With others	245(21.1%)	80(8.0%)			
With children under 16	217(18.7%)	462(46.1%)	187.4	1	< 0.001
**Source of income **(MV)	0	0			
Government or occupational pension	1050 (90.5%)	38(3.8%)	1617.5	1	< 0.001
Family transfers	54(4.7%)	305(36.4%)	347.3	1	< 0.001
Disability pension	9(0.8%)	1(0.1%)	5.3	1	0.02
Rent	0	122(12.2%)	149.7	1	< 0.001
Paid work	0	6(0.6%)	7.0	1	0.008
Have health insurance plan	14(1.2%)	769(76.9%)	1327.2	1	< 0.001
**Number of household assets^2 ^**(MV)	0	0			
0-3 assets	6(0.5%)	108(10.8%)	113.3	1	< 0.001
More than 3 assets	1154(99.5%)	894(89.2%)			

MV = Missing values

^1 ^Participants were asked 'What was your best (highest level) job?'

^2 ^household assets: television, fridge, water and electricity utilities, telephone, plumbed toilet and plumbed bathroom

All of the self-reported diagnoses and impairments, except hearing problem and limb impairment, were much less common in the rural sample (Table 2). Older people in the rural sample were four times less likely to report three or more limiting impairments and nearly five times more likely to rate their health positively. The picture was different for diagnoses made on the basis of clinical interview and examination. The prevalence of dementia was similar, while that of hypertension was just 20% lower, and that of uncontrolled hypertension 30% higher in the rural sample. Significantly more rural (22.2%) than urban (13.6%) elderly reported chronic pain. Rural residents were more likely to have smoked, and to continue to smoke, to have engaged in hazardous drinking, and to be sedentary, but were less likely to be obese.

**Table 2:** Health status in Xicheng (urban) and Daxing (rural).

**Health condition**	**Urban, Xicheng** **(n = 1160)**	**Rural, Daxing** **(n = 1002)**	**Prevalence ratio (rural vs urban) adjusted for age and gender**
**Diagnosed diseases **(MV)	0	0	
Dementia	84(7.2%)	56(5.6%)	0.96(0.70-1.32)
History of hypertension, and/or meets ESH criteria	726(62.6%)	500(49.9%)	0.80(0.74-0.87)
Uncontrolled hypertension (ESH criteria)	471(40.6%)	487(48.6%)	1.29(1.10-1.33)
ICD-10 Depression	3(0.3%)	7(0.7%)	3.05(0.83-11.2)
**Self-reported diagnoses **(MV)	1	0	
Diabetes	195(16.8%)	9(0.9%)	0.05(0.03-0.10)
Ischaemic heart disease (myocardial infarction or angina)	115(9.9%)	12(1.2%)	0.12(0.07-0.23)
Stroke	109(9.4%)	18(1.8%)	0.20(0.12-0.34)
Chronic obstructive pulmonary disease	36(3.1%)	16(1.6%)	0.54(0.30-0.96)
**Self-reported impairments **(MV)	0	0	
Arthritis	165(14.2%)	20(2.0%)	0.14(0.09-0.23)
Eye problem	194(16.7%)	65(6.5%)	0.41(0.31-0.55)
Hearing problem	142(12.2%)	86(8.6%)	0.83(0.64-1.08)
Cough problem	33(2.8%)	14(1.4%)	0.51(0.27-0.96)
Breathing problem	52(4.5%)	19(1.9%)	0.45(0.27-0.74)
Heart problem	329(28.4%)	31(3.1%)	0.11(0.08-0.16)
Gastrointestinal problem	67(5.8%)	12(1.2%)	0.22(0.12-0.40)
Fainting problem	62(5.3%)	10(1.0%)	0.19(0.10-0.37)
Limb problem	72(6.2%)	44(4.4%)	0.77(0.53-1.12)
Skin problem	12(1.0%)	2(0.2%)	0.20(0.04-0.94)
Three or more physical impairments	208(17.9%)	39(3.9%)	0.23(0.17-0.33)
Pain that interferes with life	158(13.6%)	222(22.2%)	1.60(1.31-1.94)
**Locomotion (observed) **(MV)	0	0	
Obvious abnormality of walking	53(4.6%)	27(2.7%)	0.64(0.41-1.04)
**WHODAS II disability score **(MV)	10	2	
Mean	8.1 ± 20.1	8.0 ± 14.6	1.20(0.96-.49)^1^ 0.59(0.51-0.67)^2^
**Self-rated health **(MV)	0	0	
'Good' or 'very good'	176(15.2%)	690(68.9%)	4.59(3.94-5.35)
**Dependency**	0	0	
Needs any care	183(15.8%)	54(5.4%)	0.41(0.30-0.54)
Needs much care	119(10.3%)	30(3.0%)	0.34(0.23-0.51)
**Chronic disease risk factors **(MV)	12	4	
Ever smoked	284(24.5%)	336(33.5%)	1.28(1.12-1.46)
Current smoker	193(16.6%)	305(30.4%)	1.68(1.43-1.97)
20 or more pack years of smoking	180(15.5%)	274(27.3%)	1.62(1.38-1.91)
Hazardous drinker in early life	26(2.2%)	73(7.3%)	2.89(1.83-4.52)
Current hazardous drinker	17(1.5%)	42(4.2%)	2.66(1.55-4.56)
No walks of > 0.5 km in last month	209(18.0%)	384(38.3%)	1.39(1.32-1.47)
Obesity (meets waist circumference criterion for metabolic syndrome)	530(45.7%)	158(15.8%)	0.35(0.30-0.41)

^1^Negative binomial regression

^2^Zero inflated negative binomial regression

MV = Missing values

Rural residents (6.1%) were strikingly less likely than urban residents (38.6%) to have used any health services over the three months prior to the survey. Underutilisation of services by rural residents was apparent even after controlling for age, gender and number of limiting physical impairments (Prevalence ratio 0.24, 95% CI 0.19 to 0.32). Underutilisation by rural elderly was equally apparent for primary care services (3.8% versus 20.9%, adjusted PR 0.32, 95% CI 0.23 to 0.45), hospital doctor services (2.2% versus 23.4%, adjusted PR 0.14, 95% CI 0.09 to 0.22) and hospital admission (0.5% versus 2.4%, adjusted PR 0.43, 95% CI 0.15-1.20). Hypertension was less likely to be detected among rural compared with urban residents, and detected cases were much less likely to be controlled (Table 3). The net result was that only 2.6% of all cases of hypertension were controlled in rural Daxing compared with 35.1% in urban Xicheng.

**Table 3:** Detection and control of hypertension, by site

	**Urban, Xicheng (n = 1160)**	**Rural, Daxing (n = 1002)**	***χ*^2^**	***ν***	***P***
**Detection and control of hypertension **(MV)	0	0			
The proportion of all hypertension cases that are detected	78.5%(570/726)	50.8%(254/500)	103.2	1	< 0.001
The proportion of detected cases that are treated	96.8%(552/570)	99.6%(253/254)	6.0	1	0.015
The proportion of detected cases that are controlled	44.7%(255/570)	5.1%(13/254)	125.7	1	< 0.001
The proportion of all cases that are controlled	35.1% (255/726)	2.6% (13/500)	181.5	1	< 0.001

MV = Missing values

In both urban and rural sites, numbers of physical impairments were the strongest independent predictors of health service utilisation, after controlling for age, gender, education, assets, pension availability and health insurance (Table 4). Dementia was associated with health service utilization only in rural Daxing, but the association was no longer apparent after controlling for covariates. Economic factors (household assets, receipt of pension and health insurance) predicted health service utilization only in urban Xicheng.

**Table 4:** Predictors of health service utilization (crude and adjusted robust Prevalence Ratios [PRs] with 95% confidence intervals [CI])

	**Crude PRs (95% CI)**		Adjusted PRs* (95% CI)	
		
	**Urban**	**Rural**	**Urban**	**Rural**
Dementia	1.12(0.86-1.45)	2.19(1.05-4.57)	0.94(0.73-1.20)	1.54(0.82-3.06)
Number of limiting physical illnesses				
None	1 (ref)	1 (ref)	1(ref)	1 (ref)
1-2	2.32(1.76-2.84)	4.02(2.31-7.00)	2.26(1.79-2.87)	3.82(2.12-6.85)
3 or more	3.78(2.98-4.81)	8.91(4.52-17.6)	3.74(2.94-4.75)	8.31(4.06-17.0)
Age (per 5 year increment)	1.03(0.96-1.10)	0.93(0.73-1.17)	1.00(0.96-1.08)	0.80(0.61-1.04)
Gender (male vs. female)	0.91(0.79-1.04)	0.87(0.53-1.41)	0.89(0.77-1.03)	0.99(0.58-1.70)
Education (per level)	1.04(0.99-1.09)	0.90(0.77-1.07)	1.02(0.96-1.08)	0.86(0.63-1.19)
Assets (per quarter)	1.28(1.14-1.44)	0.90(0.77-1.05)	1.23(1.09-1.38)	0.84(0.70-1.02)
Any government or occupational pension	1.51(1.10-2.08)	1.31(0.43-3.98)	1.46(1.07-1.99)	1.17(0.39-3.57)
Have health insurance	1.87(1.33-2.62)	1.75(0.90-3.41)	1.94(1.28-2.95)	1.58(0.82-3.06)

* Mutually adjusted for all other exposures in the crude model

Among the 237 participants who were rated as needing care we described levels of disability and dependency, informal care arrangements and carer strain by site (Table 5). In both settings, people with dementia were more disabled than other needing care (mean WHODAS II score 61.1, SD 30.6 versus 33.1, SD 25.7 in Xicheng; 65.5, SD 24.3, p < 0.001 versus 33.6, SD 22.7, p < 0.001 in Daxing) and more likely to be rated as needing care 'much of the time' (77% versus 57%, p = 0.005 in Xicheng; 64% versus 46%, p = 0.18 in Daxing). Carers of people with dementia spent more time assisting with basic activities of daily living (tests for trend *χ*^2 ^= 14.1, *P *= 0.001 in Xicheng, *χ*^2 ^= 9.9, *P *= 0.007 in Daxing), particularly communication, dressing, eating, grooming and toileting. Caregiver strain, measured using the Zarit Burden Interview was also higher among those caring for people with dementia (mean ZBI score 26.4, SD 20.6, p < 0.001 versus 12.1, SD 12.6, p < 0.001 in Xicheng; 17.1, SD 14.9 versus 5.3, SD 7.7 in Daxing, p < 0.001). It was therefore important to control for dementia diagnosis, as well as the age and gender of the participant when comparing care-related variables between rural and urban settings (Table 5). Adjusted analyses suggested no differences in levels of disability or dependency between rural and urban older people needing care. However, rural carers spent less time assisting with core activities of daily living, and reported lower levels of strain. Paid care was a common option in urban Beijing; one half of dependent people with dementia and slightly less than one half of all urban dependent people paid for daytime care, with a similar proportion using night time care. Only one rural family used paid daytime care. Instead, rural carers were nearly 12 times more likely to give up or cut back on work to care, and nearly three times more likely to benefit from additional informal care from friends or family.

**Table 5:** Levels of disability and dependency, informal care arrangements and carer strain (among those identified as needing care), by site

	**Xicheng (urban)** **Total** **(n = 183)**	**Daxing (rural)** **Total** **(n = 54)**	**Effect size for Rural vs. urban contrast, adjusting for age, gender and dementia status**
**Disability and dependency (in the care recipient) **(MV)	0	2	
WHODAS 12 Disability score (mean [SD])	44.2 (30.9)	50.2 (28.4)	1.7 (-6.8,10.3)^1^
Needs care 'much of the time'	119 (65.0%)	30 (55.6%)	0.81 (0.63, 1.05)^2^
**Time spent by the carer assisting with ADL **(MV)	0	0	
0 hours	28 (15.3%)	7 (13.0%)	0.52 (0.43-0.63)^3^
1-4 hours	57 (31.1%)	26 (48.1%)	
5 hours +	98 (53.6%)	21(38.9%)	
**Assistance provided for specific ADL (> one hour/day) **(MV)	0	0	
Supervision	16 (8.7%)	8 (14.8%)	1.64 (0.70-3.85)^2^
Communication	65 (35.5%)	22 (40.7%)	1.13 (0.78, 1.64)^2^
Using transport	8 (4.4%)	2 (3.7%)	0.93 (0.23, 3.75)^2^
Dressing	55 (30.1%)	10 (18.5%)	0.59 (0.33, 1.05)^2^
Eating	57 (31.1%)	15 (27.8%)	0.78 (0.50, 1.22)^2^
Grooming	57 (31.1%)	10 (18.5%)	0.55 (0.31, 1.00)^2^
Toileting	73 (39.9%)	14 (25.9%)	0.61 (0.38, 0.96)^2^
Bathing	57 (31.1%)	10 (18.5%)	0.55 (0.31, 1.00)^2^
**Caregiver Strain**	3	0	
Zarit Burden Interview Score (mean [SD])	17.9 (17.7)	11.4 (13.3)	-8.7 (-3.9, -13.5)^1^
Caregiver mental health			
SRQ-20 Score			
Mean (SD)	0.9(2.3)	1.3(2.6)	0.1(-0.6,0.8)^1^
Median (IQR	0(0,1.0)	0 (0,1.0)	
**Characteristics of main carer **(MV)	1	2	
Relationship to older person			
Spouse	71 (38.8%)	21 (38.9%)	-
Child	69 (37.7%)	21 (38.9%)	
Daughter-/son-in-law or other relative	13 (7.1%)	11 (20.4%)	
Non-relative	30 (16.4%)	1 (1.9%)	
Gender			
Female	123 (67.2%)	27 (50.0%)	1.15 (1.05, 1.27)^2^
**Care arrangements **(MV)	0	1	
Daytime paid carer	83 (45.4%)	1 (1.9%)	0.05 (0.01, 0.33)^2^
Night time paid carer	81 (44.3%)	0	-
Carer cut back on work to care	7 (3.8%)	26 (48.1%)	11.7 (5.20, 26.4)^2^
Additional informal care	13 (7.1%)	12 (22.2%)	2.78 (1.37, 5.63)^2^

Abbreviations used in the table: MV+ missing values; WHO-DAS 2.0 = World Health Organization Disability Assessment Schedule (12-item version); ADL = activities of daily living; SRQ-20 = Self Reporting Questionnaire (20 items).

^1^Mean difference from a general linear model, with 95% confidence intervals

^2^Prevalence ratios (PRs) from Poisson regression (robust 95% confidence intervals)

^3^Prevalence ratios (PRs) from ordinal regression,(robust 95% confidence intervals)

## Discussion

We carried out a comprehensive one phase survey of two catchment areas in Beijing province; Daxing's rural villages and Xicheng in the heart of Beijing city. There were relatively few non-responders, but the higher proportion in urban Xicheng (25.7%) compared with rural Daxing (4.3%) creates some potential for response bias. We applied the same catchment area sampling techniques and research protocol in both settings, and the same research group supervised the implementation of the research. Given the proximity, shared language and culture of the two sites, we believe that the comparison was apt and likely to be informative regarding the impact of contrasting infrastructure, policies, lifestyles and family structures on health outcomes and chronic disease care. However, clearly, findings from this comparison cannot be generalised to urban and rural settings in China as a whole. In particular, Daxing is less remote, and better resourced than the majority of rural locations in China. We set out to compare rural and urban samples with respect to the health status of older people, their use of health services, and their needs for informal care. For older people, these three elements are very much inter-related. Other studies that have addressed just one or other of these elements in isolation have not provided a comprehensive overview of chronic diseases, their consequences and their management, and how these might differ in urban and rural populations. However, the broad agenda for this paper has meant that we have not been able to address each topic in detail, for which more in-depth dedicated studies will be required.

Self-reported chronic disease diagnoses (diabetes, heart disease and stroke) were more prevalent in urban Xicheng than in rural Daxing. These findings are consistent with reports from previous Chinese surveys, [37-39] but need to be interpreted with caution. There may be systematic under-ascertainment in rural sites because of low levels of awareness and help-seeking, under-detection and under-treatment. Of note, hypertension and dementia, ascertained from clinical assessments in the survey, were similarly prevalent in both sites. Low levels of education may have contributed to ignorance of chronic diseases and under-reporting [11] On the other hand, the prevalence of self-reported impairments was also much lower among older people in rural Daxing, consistent with their better self-rated overall health. Also, when zero inflation was accounted for the disability score count in the rural site was 40% lower. The lesser needs for care among rural elderly, based upon global assessment by the interviewer, is again consistent with a lower prevalence of chronic disease in Daxing. However, in interpreting these differences in health perception we should bear in mind Amartya Sen's allusion to the substantial evidence that "people in states that provide more education and better medical and health facilities are in a better position to diagnose and perceive their own morbidities than the people in less advantaged states, where there is less awareness of treatable conditions (to be distinguished from "natural" states of being)" [40]. Selective mortality may be an additional explanation for the differences in health outcomes. For rural residents a 30% excess mortality is consistently observed across several data sets, from midlife onwards. The younger age and higher proportion of widows and widowers in Daxing compared with Xicheng is consistent with a difference in midlife mortality between the two populations. Unhealthier lifestyles among the rural elderly may have contributed. Consistent with our findings, a survey in Hubei Province showed higher levels of smoking and alcohol use, and much lower levels of physical activity among older people in rural compared with urban districts [41]. While our data suggests a decline in the prevalence of current smoking among older people compared with the Beijing Longitudinal Ageing Study conducted in 1991; [11] this decline was more pronounced in urban (from 48.2% to 16.6%) than in rural districts (from 43.5% to 30.4%). In summary, our data, considered in the context of other Chinese surveys, is in no way reassuring regarding the underlying health status of the Chinese rural elderly population.

The differences in our survey in the accessibility and effectiveness of the urban and rural health services were striking. In the Third Chinese National Health Services Survey, rural and urban residents with an illness in the past two weeks were equally likely to seek help from a physician; hospitalisations were less frequent among rural residents, but only among those aged 65 and over [37,42]. However, fewer than 7% of our rural sample as opposed to nearly 40% of the urban used any health service in the three months preceding the interview. In both sites, physical health was the strongest predictor of the use of health services. Our findings were not explained by the younger age and better health of rural residents. The limited availability of local health services, [38,43] rural poverty, [37,44] the lack of effective insurance cover after the collapse of the rural Cooperative Medical System, and sharp increases in charges under the new fee-for-service system [42] are all likely to be implicated. Economic factors (household assets, receipt of pension and possessing health insurance) were all independently associated with accessing healthcare in urban Xicheng, and may have explained some of the differences in help-seeking between the two sites; limited variance of these factors probably accounts for the lack of association in rural Daxing. Detection and control of hypertension is an important index of the effectiveness of community healthcare. The control of blood-pressure-related disease is a global health priority [45]. The prevalence of hypertension among older people in China has risen sharply over the period 1991-2006, [12,46,47] and prevention and control are also clear national priorities. Parameters for awareness and control in urban Xicheng were similar to those recently reported for older people in urban Chengdu, [48] while those for rural Daxing were a little worse than those from the national InterASIA survey of 2000-2001, described at that time as 'unacceptably low' [49].

Underutilisation of health services, and lack of routine medical checks may explain the low detection rates [13,22,50]. Lack of control among those who were detected and treated was a particular problem in rural Daxing. In Chengdu, [4] lack of control of hypertension was associated with infrequent blood pressure checks, under-treatment, poor treatment adherence, and ignorance of risk factors and potential complications. Hypertension in mid-life is a recognized risk factor for dementia [51-53] Therefore, the extent to which prevention and control of hypertension can be established early in the coming epidemic in China and other LAMIC may have important implications for the size of the predicted increase in numbers of people with dementia in those regions [54]. As others have noted, there is an urgent need to promote access to healthcare in China [42]. Adequate insurance or subsidy to cover health care costs, need to be extended to those outside of the urban cadres, particularly rural residents, those without formal employment and older people [18]. Community healthcare services need to be strengthened. However, attention needs also to be given to increasing the demand for healthcare; health promotion and education to encourage healthy behaviours and help-seeking [55]. Older people need to be targeted [41].

In Daxing, the burden of support and care, where it was required, fell mainly on family members who had often given up work to care. In Xicheng, family members rarely gave up work to care, paid caregivers being employed instead. These stark differences are understandable in the context of China's rapid economic development. Urban Beijing is experiencing a boom, while development in rural areas stagnates. Widening differentials in salary levels between the city and the country drive the trend towards the employment of women from less developed provinces to care for dependent older people in the city. Residential care is costlier, and associated with considerable stigma. Some caution is indicated in interpreting the higher levels of carer strain among urban compared with rural carers, since measurement bias between urban and rural settings may have been implicated; nevertheless, the finding seems plausible. Although the literature is inconsistent on this point, [56] juggling work roles with those of parent, organisational and 'hands-on' caregiver for an older relative can be stressful. In Daxing, traditional extended family living arrangements are still the norm, with neighbours and relatives available to provide additional informal care. In China, as in the Dominican Republic [35] and the USA [36] dementia is consistently associated with greater needs for care, more time spent caregiving and greater caregiver strain. Non-communicable diseases are already leading causes of mortality in China [55] and the pace of demographic ageing in China is such that predicted increases in numbers of dependent people [57], and numbers of people with dementia [54] will be greater in absolute and relative terms than for almost any other world region. Developing policies and investing in long-term care should be key priorities, alongside health sector reform.

## Conclusion

Self-reported diabetes, heart disease and stoke were more prevalent in urban Xicheng than in rural Daxing, conversely hypertension and dementia, ascertained from clinical assessments in the survey, were similarly prevalent in both sites. Apparent better health in rural Daxing might be explained by under-diagnosis (and limited access to health care facilities), under-reporting or selective mortality. Care need was common in both sites but whilst informal care was the norm in rural Daxing, paid caregivers were very common in urban Xicheng. The health reform in China should ensure access and long term care in rural settings and at the same time meet the important implications of the socio-cultural and economic changes already apparent in urban China.

## Competing interests

The 10/66 Dementia Research Group works closely with Alzheimer's Disease International (ADI), the non-profit federation of 77 Alzheimer associations around the world. ADI is committed to strengthening Alzheimer associations worldwide, raising awareness regarding dementia and Alzheimer's Disease and advocating for more and better services for people with dementia and their caregivers. ADI is supported in part by grants from GlaxoSmithKline, Novartis, Lundbeck, Pfizer and Eisai.

## Authors' contributions

MP leads the 10/66 Dementia Research Group study and CF acts as research coordinator assisted by EA and RS. SL and YH are the principal investigators in China, and ZL is the study coordinator in China. They were assisted in the conduction of the study by FY and WD. ZL and EA wrote the first draft of the paper and carried out the analyses with the assistance of MP and YH. All other authors reviewed the report and provided further contributions and suggestions. All authors approved the final manuscript.

## Pre-publication history

The pre-publication history for this paper can be accessed here:


